# Evaluation of preanalytical and postanalytical phases in clinical biochemistry laboratory according to IFCC laboratory errors and patient safety specifications

**DOI:** 10.11613/BM.2022.030701

**Published:** 2022-08-05

**Authors:** Nergiz Zorbozan, Orçun Zorbozan

**Affiliations:** 1 Kemalpaşa State Hospital, Medical Biochemistry, İzmir, Turkey; 2 Ege University Faculty of Medicine, Department of Parasitology, İzmir, Turkey

**Keywords:** total quality management, preanalytical phase, quality indicators, six sigma

## Abstract

**Introduction:**

The aim of the study was to determine the current state of laboratory’s extra-analytical phase performance by calculating preanalytical and postanalytical phase quality indicators (QIs) and sigma values and to compare obtained data according to desired quality specifications and sigma values reported by The International Federation of Clinical Chemistry and Laboratory Medicine (IFCC) Working Group – Laboratory errors and Patient Safety.

**Materials and methods:**

Preanalytical and postanalytical phase data were obtained through laboratory information system. Rejected samples in preanalytical phase were grouped according to reasons for rejection and frequencies were calculated both monthly and for 2019. Sigma values were calculated according to “short term sigma” table.

**Results:**

The number of rejected samples in laboratory was 643 out of 191,831 in 2019. Total preanalytical phase rejection frequency was 0.22%. According to the reasons for rejection, QIs and sigma values were: “Samples with excessive transportation time”: 0.0036 and 5.47; “Samples collected in wrong container” 0.02 and 5.11. In December, QIs and sigma values were: “Samples with excessive transportation time”: 0.01 and 5.34; “Samples collected in wrong container”: 0.03 and 4.98. The postanalytical QIs and sigma values were: “Reports delivered outside the specified time”: 0.34 and 4.21; “Turn around time of potassium”: 56 minute and 3.84, respectively. There were no errors in “Critical values of inpatients and outpatients notified after a consensually agreed time”.

**Conclusions:**

Extra-analytical phase was evaluated by comparing it with the latest quality specifications and sigma values which will contribute to improving the quality of laboratory medicine.

## Introduction

After decades of development, clinical laboratories have achieved a low error rate in the analytical process outpacing other processes of the total testing process (TTP), focusing on analytical quality, with standardized procedures, an internal and external quality control assessment. International accreditation bodies require laboratories to control all testing processes, focusing not only on the analytical phases but also on the preanalytical and postanalytical phase where most errors occur. Improving the extra-analytical phase of the TTP is an important responsibility for laboratory medicine (*1*).

The International Federation of Clinical Chemistry and Laboratory Medicine (IFCC) “Laboratory Errors and Patient Safety” Working Group (WG-LEPS) launched a project in 2008 to define the Model of quality indicators (QIs). The overall goal of the project is to collect standardized data and create a common reporting system for clinical laboratories based on these data. In the first phase of the project, QIs were determined for the preanalytical, analytical, and postanalytical phases, which are the main components of the TTP. The QIs result reports that are collected from participating laboratories from February 2008 to December 2009 and the preliminary quality specifications determined according to these results were published in 2011 (*2*). In 2015, Plebani *et al.* reported very high priority preanalytical QIs (*3*). In 2016, postanalytical phase QIs specifications calculated according to 2012, 2013, and 2014 data were published (*4*). Finally, the preanalytical phase and postanalytical phase QIs specifications and estimated sigma values determined according to the data collected in 2014, 2015, and the first half of 2016 were reported (*5*).

Quality indicators are one of the main tools used to increase the quality of laboratory services, ensuring patient safety by reducing error rates. Quality indicators are recognized as part of laboratory improvement strategy and have proven to be suitable tools for improving and monitoring processes (*4*).

Six Sigma is a data-driven quality strategy that provides information about process performance and is used to improve processes. The quality assessment made by this method consists of “define”, “measure”, “analyse”, “improve”, and “control” steps. In the “measure” step of the process, the number of errors is converted to the number of defects *per* million opportunities (DPMO), and the process sigma level is calculated. The “Six” in Six Sigma refers to the ideal goal where six standard deviations can fit within the defined tolerance limits of a process and anything beyond these tolerance specifications is considered a defect. The evaluation of laboratory processes with the Six Sigma method not only reduces errors that may affect patient health but also contributes positively to the healthcare institution’s budget by preventing unnecessary costs. In addition, by calculating the laboratory performance with harmonised criteria, it is possible to compare the performance with other clinical laboratories in the world (*6*).

The aim of the study was to determine the current state of our laboratory’s extra-analytical phase performance by calculating the preanalytical and postanalytical phase QIs and sigma values and to compare the obtained data according to the quality specifications and sigma values reported by the IFCC WG-LEPS.

## Materials and methods

This retrospective observational study was conducted in Hospital Central Laboratory in 2019. The data of the rejected samples in our laboratory are recorded through the “laboratory error classification system” software integrated into the laboratory information system (LIS). This software provides standardization of registration information of rejected samples. The total number of samples accepted to the laboratory, the number of rejected samples, the reasons for rejection, the total number of checked samples for haemolysis, and the total number of samples with anticoagulant checked for clots were obtained from the LIS. The haemolysis was detected by the haemolysis index of the Advia 1800 (Siemens Corp., New York, USA) autoanalyser. Clotted samples were detected by visual inspection of the specimen.

Rejected sample frequencies were calculated both monthly and for 2019. The target value for the “total preanalytical phase rejection frequency” was determined according to the average preanalytical rejection frequency values of the previous year in our laboratory (0.3%).

The rejected samples in the preanalytical phase were grouped according to the reasons for rejection. According to the data obtained, the preanalytical phase was evaluated with percentage of: “Number of samples not received / Total number of samples” (Pre-NotRec), “Number of samples collected in wrong container / Total number of samples” (Pre-WroCo), “Number of samples rejected due to haemolysis / Total number of checked samples for haemolysis” (Pre-HemR), “Number of samples clotted / Total number of samples with an anticoagulant checked for clots” (Pre-Clot), “Number of samples with insufficient sample volume / Total number of samples” (Pre-InsV), “Number of samples with inappropriate sample-anticoagulant volume ratio / Total number of samples with anticoagulant” (Pre-SaAnt), “Number of samples with excessive transportation time / Total number of samples” (Pre-ExcTim) (*7*).

The postanalytical phase was evaluated with the percentage of: “Number of reports delivered outside the specified time / Total number of reports” (Post-OutTime), “Number of critical values of inpatients notified after a consensually agreed time (from result validation to result communication to the clinician) / Total number of critical values of inpatients to communicate” (Post-InpCV), “Number of critical values of outpatients notified after a consensually agreed time (from result validation to result communication to the clinician) / Total number of critical values of outpatients to communicate” (Post-OutCV), turn around time (minutes) of: “Potassium (K) at 90th percentile (STAT)” (Post-PotTAT), “International Normalized Ratio (INR) value at 90th percentile (STAT)” (Post-INRTAT), “Troponin I (TnI) or Troponin T (TnT) at 90th percentile (STAT)” (Post-TnTAT), “time (from result validation to result communication to the clinician) to communicate critical values of inpatients (minutes)” (Post-InpCVT) and “outpatient (minutes)” (Post-OutCVT) (*5*).

Extra-analytical phase errors are assumed to be not normally distributed. Therefore, to avoid overestimating the deviation in the extra-analytical phase performance, it is recommended not to include the 1.5 standard deviation (SD) shift in the sigma calculation and to determine the DPMO values according to the short-term sigma table (6,8))). In the study, sigma values were determined according to the “short term sigma” table. Defects *per* million opportunities were calculated and converted to short term sigma (*5*, *9*). The preanalytical and postanalytical phase QIs values were calculated using the formulas recommended by the IFCC WG-LEPS (Table 1) (*5*, *8*). The calculated QIs and sigma values were evaluated, both monthly and for 2019, according to the desired (50th percentile) specifications reported by IFCC WG-LEPS (Table 1) (*5*, *7*). The sigma values were calculated according to the number of errors determined by accepting “the desired quality specification” as the target value.

**Table 1:** The QIs and sigma values of the preanalytical and postanalytical phase in our laboratory in 2019 and desired QIs and sigma values reported by IFCC WG-LEPS

**Preanalytical phase**	**Quality indicators**	**Desired Laboratory Results (50th)** **(95%Cl)**	**Desired Sigma Values (50th) (95%Cl)**	**QI (%)**	**Sigma**
Pre-NotRec	Percentage of: Number of samples not received / Total number of samples	0.19 (0.14–0.30)	4.39 (4.25–4.49)	0.01	5.22
Pre-WroCo	Percentage of: Number of samples collected in wrong container / Total number of samples	0.01 (0.004–0.01)	5.22 (5.22–5.44)	0.02*	5.11^†^
Pre-HemR	Percentage of: Number of samples rejected due to haemolysis / Total number of checked samples for haemolysis	0.44 (0.30–0.50)	4.12 (4.07–4.25)	0.22	4.36
Pre-Clot	Percentage of: Number of samples clotted / Total number of samples with an anticoagulant checked for clots	0.24 (0.20–0.27)	4.32 (4.28–4.38)	0.05	4.78
Pre-InsV	Percentage of: Number of samples with insufficient sample volume / Total number of samples	0.03 (0.02–0.04)	4.93 (4.85–5.01)	0.02	5.11
Pre-SaAnt	Percentage of: Number of samples with inappropriate sample-anticoagulant volume ratio / Total number of samples with anticoagulant	0.34 (0.22–0.42)	4.20 (4.13–4.35)	0.07	4.68
Pre-ExcTim	Percentage of: Number of samples with excessive transportation time / Total number of samples	0	6.00 (6.00–6.00)	0.0036*	5.47^†^
**Postanalytical phase**	**Quality indicators**	**Desired Laboratory Results (50th)**	**Desired Sigma Values (50th)**	**QI (%)**	**Sigma**
Post-OutTime	Percentage of: Number of reports delivered outside the specified time / Total number of reports	0.04	4.30	0.34*	4.21^†^
Post-InpCV	Percentage of: Number of critical values of inpatients notified after a consensually agreed time (from result validation to result communication to the clinician) / Total number of critical values of inpatients to communicate	1.12	3.00	0	> 6.00
Post-OutCV	Percentage of: Number of critical values of outpatients notified after a consensually agreed time (from result validation to result communication to the clinician) / Total number of critical values of outpatients to communicate	34.19	1.90	0	> 6.00
**Postanalytical phase**	**Quality indicators**	**Desired Laboratory Results (50th)**	**Desired Sigma Values (50th)**	**QI (minute)**	**Sigma**
Post-PotTAT	Turn Around Time (minutes) of Potassium (K) at 90th percentile (STAT)	49.6	ESVNA	56*	3.84
Post-INRTAT	Turn Around Time (minutes) of International Normalized Ratio (INR) value at 90th percentile (STAT)	45.0	ESVNA	36	> 6.00
Post-TnTAT	Turn Around Time (minutes) of Troponin I (TnI) or Troponin T (TnT) at 90th percentile (STAT)	53.0	ESVNA	52	4.10
Post-InpCVT	Time (from result validation to result communication to the clinician) to communicate critical values of inpatients (minutes)	NADPR	NADPR	8	> 6.00
Post-OutCVT	Time (from result validation to result communication to the clinician) to communicate critical values of outpatient (minutes)	NADPR	NADPR	10	> 6.00
*Quality indicator percentile above desired laboratory results (50^th^) (5,8). ^†^Sigma value below desired sigma values (50^th^) (5,8). ESVNA – estimate of sigma value not applicable. NADPR – not available due to poor results. IFCC WG-LEPS - The International Federation of Clinical Chemistry and Laboratory Medicine, "Laboratory Errors and Patient Safety" Working Group. Cl - confidence interval. QI - quality indicator.					

The quality specifications of the “Post-InpCVT” and “Post-OutCVT” were not reported in IFCC WG-LEPS due to insufficient results (*5*). Our target values for “Post-InpCVT” and “Post-OutCVT” are 30 minutes.

### Statistical analysis

Descriptive statistics were performed using Office 2010 Excel (Microsoft, Washington, USA) software. The results were reported as percentage (%) and number.

## Results

The number of samples received in the laboratory was 191,831 and the number of rejected samples was 643 in 2019.

Preanalytical phase: The total number of preanalytical phase errors in our laboratory was 432, the total number of checked samples for haemolysis was 130,188 and the total number of samples with an anticoagulant checked for clots was 53,504 in 2019. The total preanalytical phase rejection frequency was 0.22%. In December, the preanalytical phase rejection frequency was 0.33%. According to the reasons for rejection in 2019, “Pre-ExcTim” QIs and sigma value were 0.0036 and 5.47; “Pre-WroCo” QIs and sigma value were 0.02 and 5.11, respectively (Table 1). In December, “Pre-ExcTim” QIs and sigma value were 0.01 and 5.34; “Pre-WroCo” QIs and sigma value were 0.03 and 4.98, respectively (Table 2).

**Table 2:** The monthly QIs and sigma values of the preanalytical phase, calculated according to the reasons for rejection

**Months**		**Pre-HemR**	**Pre-InsV**	**Pre-NotRec**	**Pre-ExcTim**	**Pre-WroCo**	**Pre-SaAnt**	**Pre-Clot**
January	n	34	3	0	0	0	0	0
	N	11,936	18,617	18,617	18,617	18,617	18,617	4791
	QI (%)	0.28	0.02	0	0	0	0	0
	Sigma	4.26	5.10	> 6.00	> 6.00	> 6.00	> 6.00	> 6.00
February	n	27	1	1	1	5	0	8
	N	10,924	17,579	17,579	17,579	17,579	17,579	4272
	QI (%)	0.25	0.01	0.01	0.01*	0.03*	0	0.19
	Sigma	4.31	5.36	5.36	5.36^†^	4.95^†^	> 6.00	4.40
March	n	18	2	0	1	2	2	5
	N	11,258	18,104	18,104	18,104	18,104	18,104	4592
	QI (%)	0.16	0.01	0	0.01*	0.01*	0.04	0.11
	Sigma	4.45	5.19	> 6.00	5.37^†^	5.19^†^	4.83	4.56
April	n	16	5	1	0	3	3	2
	N	11,748	15,685	15,685	15,685	15,685	15,685	4814
	QI (%)	0.14	0.03*	0.01	0	0.02*	0.06	0.04
	Sigma	4.50	4.91^†^	5.33	> 6.00	5.05^†^	4.73	4.84
May	n	20	0	0	0	4	4	6
	N	10,186	15,487	15,487	15,487	15,487	15,487	4292
	QI (%)	0.20	0	0	0	0.03*	0.09	0.14
	Sigma	4.38	> 6.00	> 6.00	> 6.00	4.97^†^	4.61	4.49
June	n	13	1	1	0	0	5	0
	N	10,031	14,018	14,018	14,018	14,018	14,018	4174
	QI (%)	0.29	0.01	0.01	0	0	0.12	0
	Sigma	4.51	5.30	5.30	> 6.00	> 6.00	4.54	> 6.00
July	n	22	4	1	0	3	5	3
	N	11,650	13,995	13,995	13,995	13,995	13,995	4794
	QI (%)	0.19	0.03	0.01	0	0.02*	0.10	0.06
	Sigma	4.40	4.96	5.30	> 6.00	5.02^†^	4.58	4.73
August	n	20	1	0	4	0	3	0
	N	9807	12,553	12,553	12,553	12,553	12,553	4081
	QI (%)	0.20	0.01	0	0.03*	0	0.07	0
	Sigma	4.37	5.28	> 6.00	4.91^†^	> 6.00	4.68	> 6.00
September	n	28	4	4	0	1	2	0
	N	10,619	14,134	14,134	14,134	14,134	14,134	4346
	QI (%)	0.26	0.03	0.03	0	0.07*	0.05	0
	Sigma	4.29	4.95	4.95	> 6.00	5.30^†^	4.81	> 6.00
October	n	20	0	6	0	2	6	0
	N	9801	16,958	16,958	16,958	16,958	16,958	4092
	QI (%)	0.20	0	0.04	0	0.01*	0.15	0
	Sigma	4.37	> 6.00	4.89	> 6.00	5.18^†^	4.47	> 6.00
**Months**		**Pre-HemR**	**Pre-InsV**	**Pre-NotRec**	**Pre-ExcTim**	**Pre-WroCo**	**Pre-SaAnt**	**Pre-Clot**
November	n	22	4	4	0	5	7	3
	N	11,034	18,650	18,650	18,650	18,650	18,650	4635
	QI (%)	0.20	0.02	0.02	0	0.03*	0.15	0.07
	Sigma	4.38	5.02	5.02	> 6.00	4.96^†^	4.47	4.72
December	n	40	4	1	1	4	2	1
	N	11,194	16,051	16,051	16,051	16,051	16,051	4621
	QI (%)	0.36	0.03	0.01	0.01*	0.03*	0.04	0.02
	Sigma	4.19	4.98	5.34	5.34^†^	4.98^†^	4.83	5.02
2019	n	280	29	19	7	29	39	28
	N	130,188	191,831	191,831	191,831	191,831	191,831	53,504
	QI (%)	0.22	0.02	0.01	0.0036*	0.02*	0.07	0.05
	Sigma	4.36	5.11	5.22	5.47^†^	5.11^†^	4.68	4.78
*Quality indicator percentile above 50th percentile according to 2018 data of IFCC WG-LEPS (8). ^†^Sigma value below 50th percentile according to 2018 data of IFCC WG-LEPS (8). Pre-HemR – percentage of: Number of samples rejected due to haemolysis / Total number of checked samples for haemolysis. Pre-InsV – percentage of: Number of samples with insufficient sample volume / Total number of samples. Pre-NotRec – percentage of: Number of samples not received / Total number of samples. Pre-ExcTim – percentage of: Number of samples with excessive transportation time / Total number of samples. Pre-WroCo – percentage of: Number of samples collected in wrong container / Total number of samples. Pre-SaAnt – percentage of: Number of samples with inappropriate sample-anticoagulant volume ratio / Total number of samples with anticoagulant. Pre-Clot – percentage of: Number of samples clotted / Total number of samples with an anticoagulant checked for clots. N – total number of samples (The total number of checked samples for haemolysis and the total number of samples with an anticoagulant checked for clots in the columns Pre-HemR and Pre-Clot were listed). IFCC WG-LEPS - The International Federation of Clinical Chemistry and Laboratory Medicine, "Laboratory Errors and Patient Safety" Working Group. QI - quality indicator.								

Postanalytical phase: “Post-OutTime” QIs and sigma value were 0.34 and 4.21; “Post-PotTAT” QIs (minute) and sigma value were 56 and 3.84, respectively. “Post-INRTAT”, Post-TnTAT, “Post-InpCVT and “Post-OutCVT QIs (minute) were 36, 52, 8 and 10, respectively (Table1). ”Post-TnTAT” QIs (minute) in January, February, March, June and November were above the desired annual target value (Table 3). There were no errors in “Post-InpCV” and “Post-OutCV” in 2019 and all months (Table 4).

**Table 3:** The QIs and sigma values of “Post-OutTime”, “Post-PotTAT”, “Post-INRTAT” and “Post-TnTAT”

**QIs**		**January**	**February**	**March**	**April**	**May**	**June**	**July**	**August**	**September**	**October**	**November**	**December**	**2019**
Post-OutTime	QI (%)	0.18*	0.14*	0.41*	0.36*	0.26*	0.36*	0.18*	0.46*	0.35*	0.17*	0.23*	0.80*	0.34*
	Sigma	4.42	4.50	4.14^†^	4.18^†^	4.31	4.19^†^	4.42	4.10^†^	4.19^†^	4.44	4.34	3.91^†^	4.21^†^
Post-PotTAT	QI (minute)	55*	52*	55*	54*	59*	59*	54*	58*	61*	54*	55*	57*	56*
	Sigma	3.97	3.68	3.44	4.05	4.44	4.09	3.75	3.76	3.93	> 6.00	3.65	3.85	3.84
Post-INRTAT	QI (minute)	34	36	35	39	41	38	35	35	36	34	37	35	36
	Sigma	> 6.00	> 6.00	> 6.00	> 6.00	> 6.00	> 6.00	> 6.00	> 6.00	> 6.00	> 6.00	> 6.00	> 6.00	> 6.00
Post-TnTAT	QI (minute)	59*	60*	54*	46	46	55*	50	50	51	51	54*	49	52
	Sigma	4.28	3.67	4.27	4.08	4.27	4.08	> 6.00	4.33	4.30	3.75	4.00	> 6.00	4.10
*Quality indicator percentile above 50^th^ percentile according to 2014 data of IFCC WG-LEPS (5). ^†^Sigma value below 50^th^ percentile according to 2014 data of IFCC WG-LEPS (5). Post-OutTime – percentage of: Number of reports delivered outside the specified time / Total number of reports. Post-PotTAT – turn Around Time (minutes) of Potassium (K) at 90th percentile (STAT). Post-INRTAT – turn Around Time (minutes) of International Normalized Ratio (INR) value at 90th percentile (STAT). Post-TnTAT – turn Around Time (minutes) of Troponin I (TnI) or Troponin T (TnT) at 90th percentile (STAT). DPMO – defects *per* million opportunities. QI – quality indicators. IFCC WG-LEPS - The International Federation of Clinical Chemistry and Laboratory Medicine, "Laboratory Errors and Patient Safety" Working Group.														

**Table 4:** QIs and sigma values of “Post-InpCV”, “Post-OutCV”, “Post-InpCVT” and “Post-OutCVT”

**QIs**		**January**	**February**	**March**	**April**	**May**	**June**	**July**	**August**	**September**	**October**	**November**	**December**	**2019**
Post-InpCV	QI (%)	0	0	0	0	0	0	0	0	0	0	0	0	0
	Sigma	> 6.00	> 6.00	> 6.00	> 6.00	> 6.00	> 6.00	> 6.00	> 6.00	> 6.00	> 6.00	> 6.00	> 6.00	> 6.00
Post-OutCV	QI (%)	0	0	0	0	0	0	0	0	0	0	0	0	0
	Sigma	> 6.00	> 6.00	> 6.00	> 6.00	> 6.00	> 6.00	> 6.00	> 6.00	> 6.00	> 6.00	> 6.00	> 6.00	> 6.00
Post-InpCVT (minute)	/	8.81	4.20	7.72	7.90	8.42	3.02	5.19	5.80	9.54	10.05	13.18	6.61	8.00
Post-OutCVT (minute)	/	16.47	6.76	8.24	9.09	6.89	9.22	8.29	5.90	7.90	12.20	16.27	7.93	10.00
Post-InpCV – percentage of: Number of critical values of inpatients notified after a consensually agreed time (from result validation to result communication to the clinician) / Total number of critical values of inpatients to communicate. Post-OutCV – percentage of: Number of critical values of outpatients notified after a consensually agreed time (from result validation to result communication to the clinician) / Total number of critical values of outpatients to communicate. Post-InpCVT – time (from result validation to result communication to the clinician) to communicate critical values of inpatients (minutes). Post-OutCVT – time (from result validation to result communication to the clinician) to communicate critical values of outpatient (minutes). QI – quality indicators.														

## Discussion

According to the data of our study, the “total pre-analytical phase errors” sigma value was 4.34 in 2019. In 2019, the “Pre-ExcTim” and “Pre-WroCo” QIs and sigma values were unacceptable according to the desired specifications reported by the IFCC WG-LEPS. When QIs and sigma values were calculated based on monthly data, “Pre-ExcTim” was unacceptable in February, March, August, December, and “Pre-WroCo” was unacceptable in months except for January, June and August according to the annual target value.

Document ISO 15189: 2012 recommends monitoring all critical aspects of the TTP and comparing it with data entered by different laboratories, taking into account all events that caused a particular error (*10*).

There is no monthly or annual target value for “total preanalytical phase rejection frequency” in the IFCC WG-LEPS (*7*). In our laboratory, we begin the preanalytical phase evaluation by comparing the monthly “total preanalytical phase rejection frequency” with the “average of total preanalytical phase rejection frequency of the previous year”. If the monthly total preanalytical phase rejection frequency is higher than the average of the previous year, we group them according to the reasons for rejection, then evaluate the QIs and sigma values according to the annual desired target values reported by IFCC WG-LEPS. This approach, in which we evaluate our preanalytical phase data monthly before making the annual evaluation, provides us with an early intervention opportunity for error sources. It also prevents the cumulative accumulation of errors. In December, the “total preanalytical rejection frequency” was higher than “the average preanalytical rejection frequency value of the previous year of our laboratory”. When we evaluated December’s data according to the reasons for rejection, “Pre-ExcTim” and “Pre-WroCo” were unacceptable according to the annual target value.

In the postanalytical process evaluation, the “Post-OutTime” QIs and sigma values were unacceptable. Based on monthly data, the “Post-OutTime” QIs value was unacceptable in all months, the “Post-OutTime” sigma values were unacceptable in March, April, June, August, September and December according to the annual desired target value. When we examined our data in detail for the implementation of the regulatory preventive action, we saw that the reports delivered outside the specified time were clustered on certain days. It was determined that the number of reports delivered outside the specified period increased due to the device failure in March and April, and the need for extra maintenance during June, August, September, and December.

Shewhart divides the source of variability in processes into two groups as general (chance causes, common causes) and special (assignable causes, special causes) reasons. While general causes are always emerging and predictable, specific causes occur in few numbers and have significant effects on their own (*11*). Device breakdown and the need for extra device maintenance are special sources of variation (*11*, *12*). In laboratories with more than one auto-analyser, the reporting times do not change significantly during a device failure or device maintenance, as the tests can be analysed with another auto-analyser that functions. However, for laboratories that have only one autoanalyser, device malfunctions and unforeseen maintenance requirements are important time-related error sources. For this reason, it may be beneficial to present the data obtained in the studies for the harmonization of quality specifications according to subgroups by considering the capacities of the laboratories or the number of devices.

Our “Post-PotTAT” QIs value was unacceptable. When the data were evaluated monthly, the “Post-PotTAT” QIs in all months were unacceptable relative to the annual target value. The “Post-TnTAT” QIs value was acceptable relative to the target value. However, when evaluated monthly, “Post-TnTAT” in January, February, March, June, and November was unacceptable compared to the annual target. The sigma values of the “Post-PotTAT”, “Post-INRTAT” and “Post-TnTAT” are not determined in the IFCC WG-LEPS report, because they could not be expressed as a percentage (*5*).

The maximum time target for critical value notifying is 30 minutes in our laboratory. With the software we added to our LIS, the system sends an information message to the users’ mobile phones when there is a critical value in the report. This software has prevented errors for the “Post-InpCV” and the “Post-Out CV”.

In conclusion, in this study, in which we evaluated the extra-analytical phase of our laboratory, the “Pre-ExcTim”, the “Pre-WroCo” and the “Post-PotTAT” QIs were unacceptable. Laboratory medicine will become a safer diagnostic discipline in health through error reduction strategies that are a routine part of quality management programs implemented in clinical laboratories. In this direction, we think that the approach followed in our study, in which the extra-analytical phase is evaluated by comparing it with the latest quality specifications and sigma values published by IFCC WG-LEPS will contribute to the improving the quality of laboratory medicine.
